# Severe pulmonary alveolar proteinosis with respiratory failure treated by intrapulmonary percussive ventilation

**DOI:** 10.1002/rcr2.676

**Published:** 2020-11-04

**Authors:** Takahiro Tashiro, Yusuke Tomita, Megumi Inaba, Kumiko Hayashi, Naomi Hirata, Takuro Sakagami

**Affiliations:** ^1^ Department of Respiratory Medicine Kumamoto Chuo Hospital Kumamoto Japan; ^2^ Department of Respiratory Medicine, Graduate School of Medical Sciences Kumamoto University Kumamoto Japan; ^3^ Department of Clinical Engineering Services Kumamoto Chuo Hospital Kumamoto Japan

**Keywords:** Foamy macrophages, intrapulmonary percussive ventilation, pulmonary alveolar proteinosis, respiratory failure, whole lung lavage

## Abstract

Pulmonary alveolar proteinosis (PAP) is a rare disease characterized by abnormal accumulation of surfactant in the alveoli. Whole lung lavage (WLL) is the standard treatment for severe autoimmune PAP (aPAP); however, it is highly invasive. Intrapulmonary percussive ventilation (IPV) is a non‐invasive technique that delivers small bursts of high‐flow respiratory gas into the lung and mobilizes secretions. As IPV is beneficial for chronic respiratory diseases such as cystic fibrosis and bronchiectasis to reduce sputum, it was hypothesized that IPV will ameliorate aPAP by mobilizing and removing accumulated surfactant and foamy macrophages. Here, we report the case of a 52‐year‐old female with severe aPAP and progressive respiratory failure. She received intermittent IPV therapy for six months and thereby showed improvement in assessments of chest computed tomography (CT), lung function, and oxygenation. We suggest that IPV should be used as an alternative therapy for patients with aPAP and respiratory failure.

## Graphical abstract

We report the first case of severe autoimmune pulmonary alveolar proteinosis (aPAP) with progressive respiratory failure successfully treated by intrapulmonary percussive ventilation (IPV) therapy for six months.

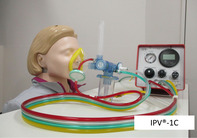

## Introduction

Pulmonary alveolar proteinosis (PAP) is a rare disease characterized by alveolar accumulation of surfactant composed of proteins and lipids due to defective surfactant clearance by alveolar macrophages [1, 2]. The current standard of care for moderate to severe autoimmune PAP (aPAP) is whole lung lavage (WLL), which requires general anaesthesia [1, 2, 3]. However, WLL is highly invasive with the risk of low oxygen saturation and pneumothorax [3]. Thus, new treatment strategies for aPAP are urgently required.

Intrapulmonary percussive ventilation (IPV) (IPV®‐1C; Percussionaire Corp., USA) is a technique that delivers small bursts of high‐flow respiratory gas into the lung, intended to mobilize secretions in several respiratory diseases [4]. These high‐frequency gas pulses expand the lungs, vibrate and enlarge the airways, and deliver gas into distal lung units beyond the accumulated mucus [4]. In addition, IPV can be performed through a face mask without general anaesthesia repeatedly. Thus, we hypothesized that IPV therapy will ameliorate aPAP by mobilizing and removing accumulated surfactant and foamy macrophages.

Here, we report the first case of severe aPAP with progressive respiratory failure successfully treated by IPV therapy for six months.

## Case Report

A 52‐year‐old woman, showing abnormal shadows on chest X‐ray, visited our hospital complaining of cough, sputum, and dyspnoea. The patient had a 30 pack‐year history of cigarette smoking. Krebs von den Lungen‐6 (KL‐6), surfactant protein D (SP‐D), and lactate dehydrogenase (LDH) serum levels were increased: LDH 310 IU/L, KL‐6 4130 U/mL, SP‐D 466 ng/mL. Arterial blood gas analysis with room air showed type 1 respiratory failure (partial pressure of arterial oxygen (PaO_2_) of 51 mmHg and partial pressure of carbon dioxide (PaCO_2_) of 41 mmHg) and abnormal alveolar‐arterial oxygen partial pressure difference (A‐aDO_2_) of 47 mmHg. Pulmonary function testing demonstrated a restrictive pattern (vital capacity of 1.57 L, 59.5% of predicted) and decreased diffusion capacity for carbon monoxide (DL_CO_; 7.85 mL/min/mmHg). Her bronchoalveolar lavage fluid contained large amounts of granular acellular eosinophilic proteinaceous material with morphologically abnormal foamy macrophages engorged with diastase‐resistant Periodic Acid Schiff PAS‐positive intracellular inclusions. Lung biopsy obtained by video‐assisted thoracoscopic surgery showed alveoli filled with eosinophilic lipoproteinaceous material with preserved lung architecture consistent with PAP. Anti‐granulocyte macrophage‐colony stimulating factor antibody concentration increased to 77.3 μg/mL. She had no evidence of autoimmune or haematological diseases. Furthermore, there was no history of exposure to mineral particles (silica, talc, cement, and kaolin) or metal particles (aluminium, titanium, and indium), which are known to trigger secondary PAP. Consequently, the patient was diagnosed with aPAP. She was prescribed long‐term oxygen therapy and ambroxol hydrochloride, and WLL was proposed for her severe aPAP. However, she refused to receive WLL.

**Figure 1:**
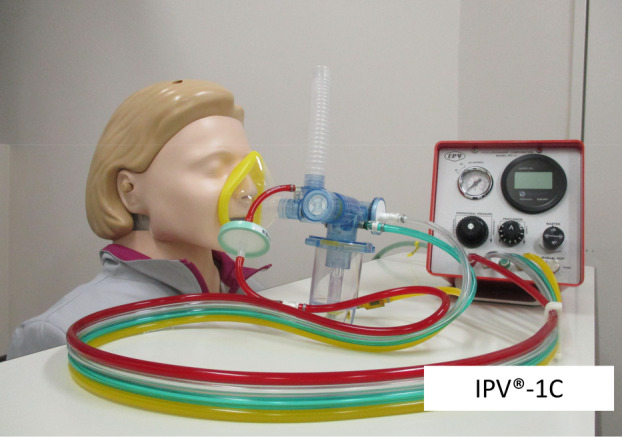
Intrapulmonary percussive ventilation (IPV) therapy. This figure shows IPV used in this study and the patient undergoing IPV therapy.

Two years after the initial diagnosis of aPAP, her chronic respiratory failure progressed and she was admitted to our hospital. She had severe hypoxaemia and low‐grade fever (37.6°C). White blood cell counts and C‐reactive protein level were 2700/μL and 40.0 mg/dL, respectively. Subsequently, high‐flow nasal canula therapy was started. Antibiotics, tazobactam/piperacillin and levofloxacin, were intravenously administered for six days after admission. To improve oxygenation, IPV was performed on the fifth day after admission (Fig. 1).

**Figure 2:**
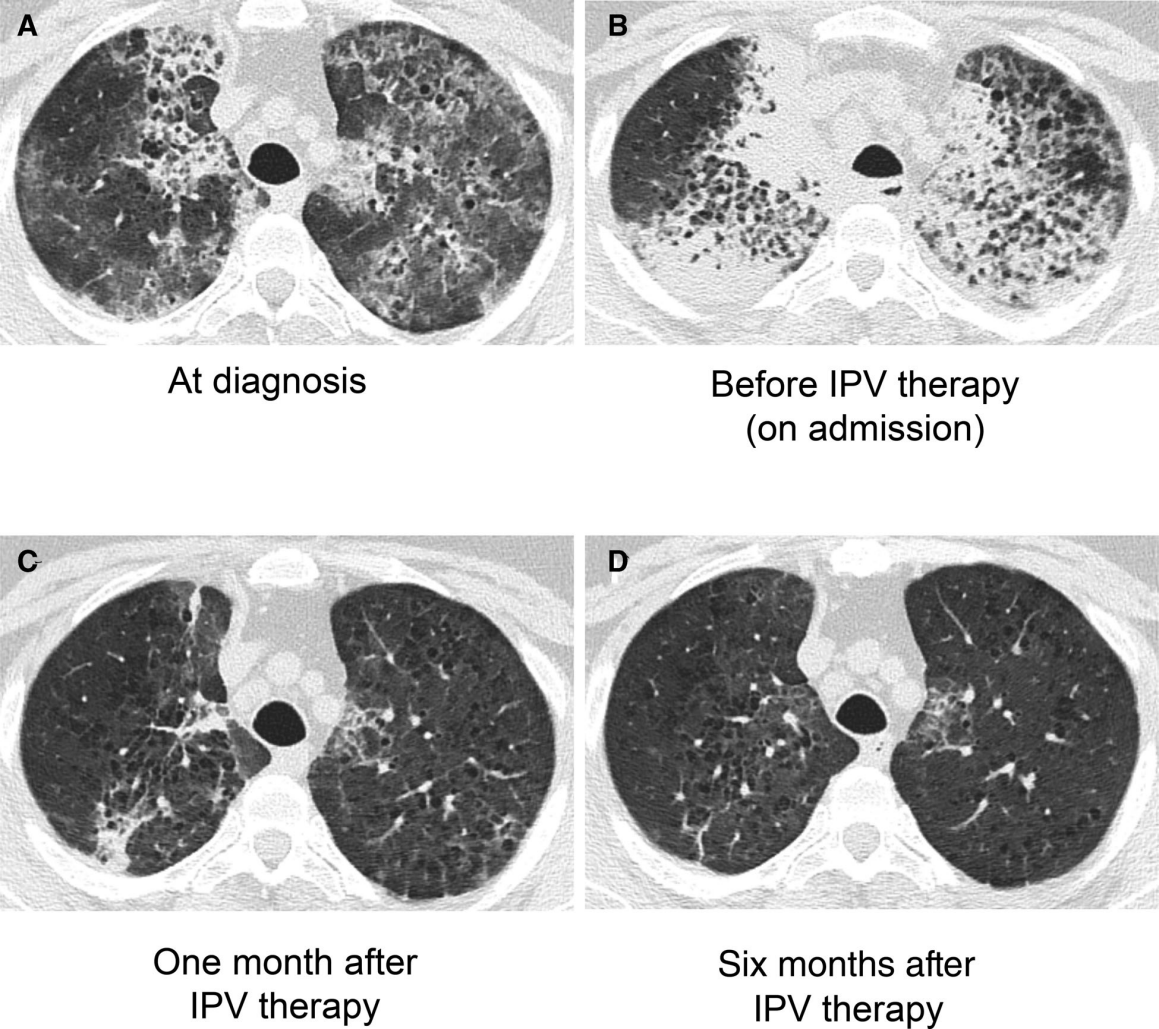
Key imaging. Computed tomography (CT) images at diagnosis (A), before intrapulmonary percussive ventilation (IPV) therapy (on admission; B), one month after IPV therapy (C), and six months after IPV therapy (D) are shown.

IPV was performed through a face mask. The percussion frequency and the peak pressure were set to 300/min and 30 pound‐force per square inch (psi), respectively. IPV was performed twice daily for 15 min each time while she was hospitalized. The amount of sputum production was increased after starting IPV therapy. Her conditions improved and she was discharged on the 16th day after admission. IPV for 15 min was continued two to three times per week for six months, at the end of which the patient achieved significant improvement in radiographic findings (Fig. 2) and respiratory condition (vital capacity 1.89 L, DL_CO_ 11.13 mL/min/mmHg, PaO_2_ with room air 69.4 mmHg, A‐aDO_2_ 25.7 mmHg). No adverse events associated with IPV were identified.

## Discussion

The current standard treatment for severe aPAP is WLL [1, 2]. However, hospitalization and general anaesthesia are required to perform WLL. The most frequent complications associated with WLL are fever, hypoxaemia, and pneumothorax [2, 3]. In some severe cases, extracorporeal oxygenation, which is a highly invasive technique, is required to maintain oxygenation during WLL therapy [3]. WLL is effective in 85% of patients, but half of the patients need to repeat the WLL therapy [2].

IPV is a non‐invasive ventilatory technique and does not require hospitalization or general anaesthesia. High‐frequency gas pulses generated by IPV expand the lungs and vibrate and enlarge the airways, thus IPV can deliver gas into distal lung units beyond the accumulated mucus in airway [4]. IPV has been reported to reduce sputum and improve atelectasis in various pathological conditions such as chronic obstructive pulmonary disease and Duchenne muscular dystrophy [4]. However, the usefulness of IPV for aPAP with respiratory failure has not been reported to date. In this case, 15 min of IPV therapy for six months significantly improved findings of chest computed tomography (CT), lung function tests, and oxygenation. We speculate that high‐frequency gas pulses generated by IPV might have mobilized and removed accumulated surfactant in lungs the amorphous substances accumulated in the airway, which resulted in improved clinical findings.

Spontaneous improvement of radiographic findings has been seen in 8% of patients with mild PAP [1]. Another review reported that spontaneous remission cases tended to have a shorter duration of symptoms before diagnosis (median four months) [5]. Although the current case was of severe aPAP and had longer duration of symptoms, we cannot exclude the possibility of spontaneous improvement of aPAP.

In conclusion, the present study describes the first case of severe aPAP with respiratory failure successfully treated by IPV therapy. We suggest that IPV could be an alternative therapy for severe aPAP with respiratory failure, particularly in cases where the WLL cannot be used.

### Disclosure Statement

Appropriate written informed consent was obtained for publication of this case report and accompanying images.
